# Acute bronchodilator responses decline progressively over 4 years in patients with moderate to very severe COPD

**DOI:** 10.1186/s12931-014-0102-5

**Published:** 2014-08-31

**Authors:** Donald P Tashkin, Ning Li, Eric C Kleerup, David Halpin, Bartolome Celli, Marc Decramer, Robert Elashoff

**Affiliations:** Department of Medicine, David Geffen School of Medicine at UCLA, 10833 Le Conte Ave, Los Angeles, CA 90095 USA; Royal Devon and Exeter Hospital, Barrack Road, Exeter, EX2 5DW UK; Brigham and Women’s Hospital, 75 Francis St, Boston, MA 02115 USA; University of Leuven, Herestraat 49, B-3000 Leuven, Belgium; Department of Biomathematics, David Geffen School of Medicine at UCLA, 10833 Le Conte Ave, Los Angeles, CA 90095 USA

**Keywords:** COPD, Bronchodilator response, UPLIFT trial

## Abstract

**Background:**

We previously reported a progressive decline in absolute responses of FEV_1_ and FVC to a near-maximal dose of 2 different short-acting bronchodilators over 4 years. Since varying host factors and the method of expressing the response may impact the time trend of acute bronchodilator responses, we now examined the potential influence of salient host characteristics on changes in bronchodilator responses over time expressed in different ways.

**Methods:**

As part of the 4-year, placebo-controlled Understanding Potential Long-term Impacts on Function with Tiotropium (UPLIFT) trial, pre- and post-bronchodilator spirometry was performed at baseline and 1 month and every 6 months thereafter. Post-bronchodilator values for FEV_1_ and FVC were analyzed for subjects completing at least the 1 year visit (Placebo – N = 2463; Tiotropium – N = 2579), stratified by GOLD stage, age, gender and smoking status and expressed as absolute, relative (%) and % predicted changes from pre-bronchodilator values. Annual changes in bronchodilator response were estimated using linear mixed effects models.

**Results:**

For all subjects analyzed, FEV_1_ and FVC bronchodilator responses showed progressive and highly significant (p < 0.0001) declines over 4 years. Declines were generally larger in patients with severe/very severe than mild/moderate airflow obstruction, in older patients (≥65 yrs) and in former than continuing smokers.

**Conclusion:**

Acute FEV_1_ and FVC responses to bronchodilators decline significantly over time in COPD patients, whether expressed as absolute, relative or % predicted changes, potentially impacting on the clinical responses to bronchodilator therapy as well as on the annual rate of decline in post-bronchodilator lung function.

**ClinicalTrials.gov number:**

NCT00144339

**Electronic supplementary material:**

The online version of this article (doi:10.1186/s12931-014-0102-5) contains supplementary material, which is available to authorized users.

## Introduction

Bronchodilator responsiveness is a well-described feature of both asthma and COPD. While the response to a bronchodilator in COPD is never complete, nonetheless it often fulfills the currently accepted criteria for a significant response [1–3], although the degree of response (and the attainment of a significant response) is highly variable between testing sessions [4,5]. Since COPD is usually a progressive disease characterized by a variably accelerated annual rate of decline in lung function, as determined from measurements of both pre- and post-bronchodilator forced expired volume in 1 sec (FEV1) [6], it is possible that the response to a bronchodilator might also change over an extended period of time; however, few studies have examined the long-term course of responses to a bronchodilator in COPD with varying results [7–9].

We recently compared the annual rates of change in the pre- versus post-bronchodilator FEV1 and FVC over 4 years in 5041 COPD UPLIFT trial participants [10] and observed that, on average, the absolute FEV1 and forced vital capacity (FVC) responses to a bronchodilator *decreased* progressively and significantly over the 4-year course of the trial, in contrast to findings from previous studies of 1-11 years duration in which either no change, small average changes of varying significance or substantial increases in responses were observed [7-9]. These differences could be due to several factors, including differences in the study populations, especially regarding the severity of airflow obstruction, as well as differences in the methods used to measure the bronchodilator response. Such methods included a standard therapeutic dose of a beta-agonist followed only 10 minutes later by repeat spirometry in the IPPB trial [7] and the Lung Health Study [8] and 400 mcg salbutamol with repeat spirometry after only 15 minutes in the Evaluation of COPD Longitudinally to Identify Predictive Surrograte Endpoints (ECLIPSE) study [9], compared to double doses of both a beta-agonist and a muscarinic antagonist and performance of the post-bronchodilator spirometry at the expected time of peak or near-peak action of each of the two classes of bronchodilators in the UPLIFT trial [11]. It is not unlikely, therefore, that the responses to a bronchodilator were sub-maximal in the earlier trials and near-maximal in the UPLIFT study.

Because of these differences in methodology for measuring the response to a bronchodilator and the possibility that varying host factors (including gender, age, severity of airflow obstruction, smoking status and use of inhaled corticosteroids) may impact the bronchodilator response over time, we extended our analysis of the time trend of bronchodilator responses (for both FEV1 and FVC) over the 4 years of the UPLIFT trial to examine the potential influence of these host factors on the changes over time in bronchodilator responses expressed in three different ways: absolute change in ml, percent change from baseline and change in percent predicted.

## Methods

We performed a post-hoc analysis of data from the UPLIFT trial in which 5993 patients with moderate to very severe COPD (mean age 65±8 yrs; mean post-bronchodilator FEV_1_ 1.32±0.44 L, 48% predicted) were randomized to receive tiotropium 18 mcg Handihaler once daily vs. placebo over a 4-year period. Detailed methods and the main results of UPLIFT have been published previously [11,12]. Briefly, as part of this trial, pre- and post-bronchodilator spirometry was performed in accordance with American Thoracic Society guidelines [13] at baseline and 1 month and every 6 months following randomization over 4 years. Identical spirometric equipment and study-specific software were used at each site with central quality-assurance review of all spirometry data throughout the trial [12,14]. At each visit, immediately following the pre-bronchodilator spirometry, patients received study drug (either tiotropium or placebo) followed by 4 inhalations of ipratropium, 18 μg/inhalation, followed 1 hour later by 4 inhalations of albuterol, 100 μg/inhalation, followed 30 min later by spirometry again. At each spirometry visit beginning at the baseline visit, study drug was also administered immediately following the pre-bronchodilator measurement. Prior to each spirometry visit, patients were instructed to withhold their respiratory medications for an appropriate period (Additional file 1). Visits were postponed if patients experienced an exacerbation within the preceding 6 weeks. The original UPLIFT trial protocol had been approved by the ethics committee at each center, and all patients had provided written informed consent. UPLIFT was a global trial involving 37 countries and 490 investigational centers. The trial was approved by the designated institutional review board at each of the participating centers.

### Analytic methods

Acute bronchodilator responses for both FEV1 and FVC were expressed as absolute (ml), relative (%) and % predicted changes from the pre-bronchodilator values. Since we noted a substantial decline in the absolute response in the tiotropium group between the baseline and 1-month assessment, responses were analyzed beginning at the 1-month assessment for patients who completed at least the 1-year post-randomization visit (N = 2463 placebo and 2579 tiotropium patients) over the 4 years of the trial. Since tiotropium resulted in a sustained increase in the pre-bronchodilator FEV1 (which could have impacted on the response to the two short-acting bronchodilators) and was administered along with the latter to assess the acute bronchodilator response in patients in the tiotropium arm of the trial, data from the placebo and tiotropium treatment groups were analyzed separately. Responses were also stratified by GOLD grading for airflow obstruction (grades I/II, III and IV), age (≤65 yrs, >65 yrs; median age was 65 yrs), gender. smoking status (continuing smokers, sustained former smokers, intermittent smokers) and use of inhaled corticosteroids (ICS) at baseline. Longitudinal analysis was conducted to estimate the annual changes in bronchodilator response over the period from the 1-month assessment to the end of the four-year follow-up. In particular, the analysis was performed using linear mixed effects models which included the subject-specific trajectories and data clustering due to repeated measures within patients. A linear time trend was assumed to describe the trajectory of bronchodilator response over the 4 year study period. The linearity assumption was tested by including a quadratic time effect, but it was not significant in the models. As an output of the model, the annual change was expressed as the estimated fixed effect of time (in years) and the standard error of this regression coefficient. Since this quantity was estimated based on a statistical model, not raw data, its estimated variability could only be expressed as standard error, not standard deviation.

Because of the possibility that changes in bronchodilator response over time within individual patients might influence the change in their health-related quality of life, we also examined, at the patient level, the relationship between decline in bronchodilator response and change in the St. George’s Respiratory Questionnaire (SGRQ) total score over the course of the study in the placebo group using a linear regression model. The outcome was change in SGRQ total score at the end of the study as compared to the baseline and the primary predictor was the patient level change in bronchodilator response per year estimated from the linear mixed effects model, adjusting for baseline % predicted FEV1 and frequency of exacerbations in the first year (> = 2 versus < 2).

Within-patient variability was expressed as the square-root of the variance for the regression residuals estimated from the linear mixed effects models, and between- patient variability in annual changes was expressed as the square-root of the variance of the slopes. Proportions of patients with a significant positive response according to ATS/ERS criteria [1], namely an increase of FEV1 and/or FVC of 12% and 200 ml above baseline, were determined at each visit and the odds ratios for annual changes in these proportions were estimated using generalized estimating equations.

## Results

**Figure 1:**
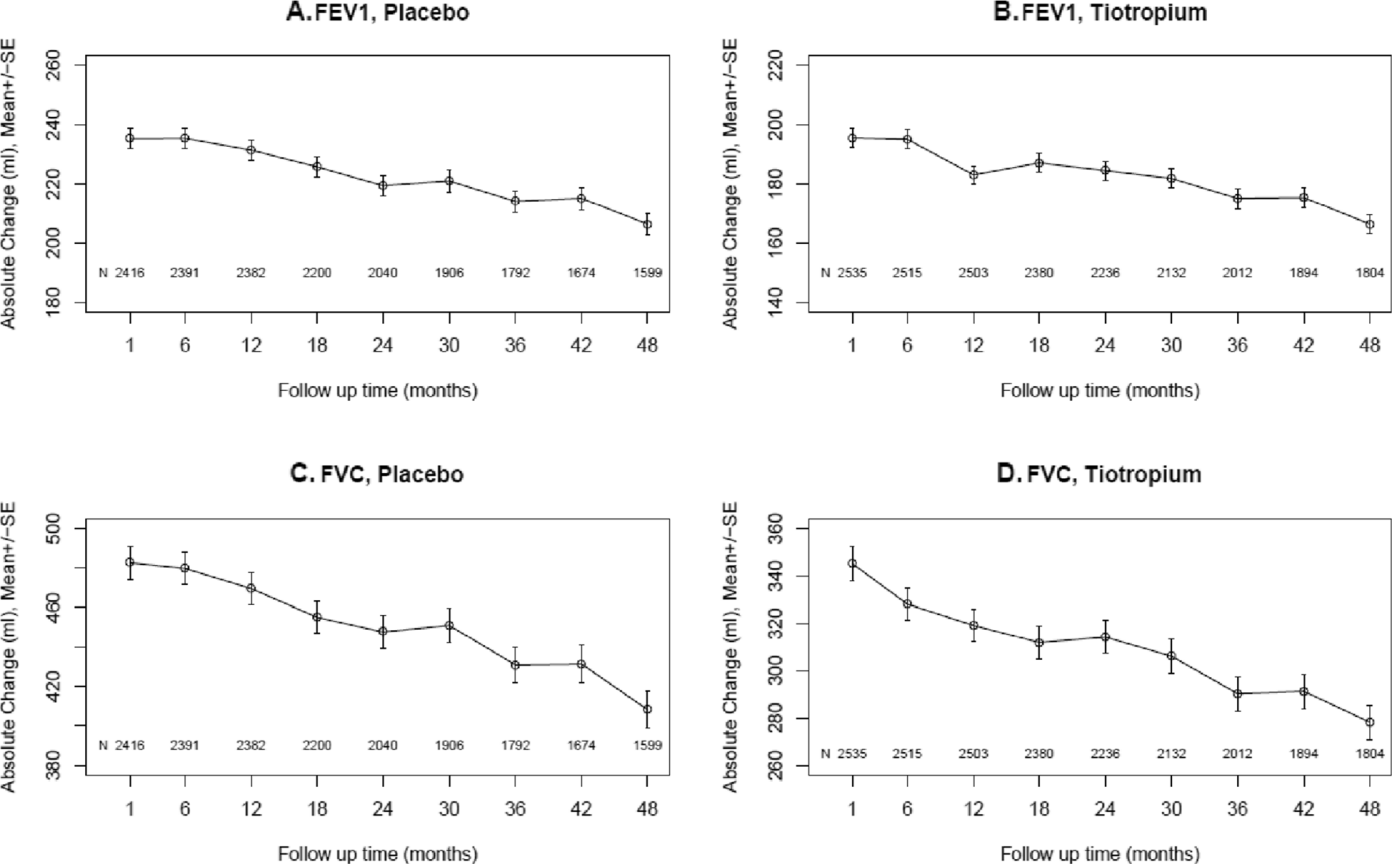
**Mean absolute bronchodilator responses (±SD) (Δ = post- minus pre-bronchodilator FEV1 and FVC, ml) over 4 years by treatment group separately (placebo and tiotropium).** Mean absolute FEV1 responses are shown for the placebo group **(A)** and the tiotropium group **(B)** separately. Mean absolute FVC responses are shown for the placebo group **(C)** and the tiotropium group **(D)** separately.

Baseline clinical characteristics of the subjects included in the analysis are shown in Table 1 for the placebo and tiotropium groups separately. These are similar to those in the entire UPLIFT population, as previously reported [11]. Mean absolute FEV1 and FVC responses to the bronchodilators (in ml ± SD) are shown for all patients, in the placebo and tiotropium groups separately, at each time point over the 4 years of the study in Figure 1. Progressive declines in both FEV1 and FVC responses are observed beginning 1 to 1½ years after the start of the study in both treatment groups.

**Table 1:** **Baseline characteristics of subjects in the placebo and tiotropium arms of the UPLIFT trial included in the analysis of time trends in bronchodilator responses over the course of the trial**

**Characteristic**	**Placebo (N = 2463)**	**Tiotropium (N = 2579)**
Age, yrs, mean (SD)	64.2 (8.39)	64.3 (8.39)
>65 yrs, N (%)	1158 (47.0%)	1223 (47.4%)
≤65 yrs, N (%)	1305 (53.0%)	1356 (52.6%)
Gender, male, N (%)	1852 (75.2%)	1965 (76.2%)
Pre-bronchodilator FEV_1_, L, mean (SD)	1.12 (0.40)	1.11 (0.40)
Pre-bronchodilator FEV_1_, % predicted, mean (SD)	39.9 (11. 8)	39.8 (11.9)
Post-bronchodilator FEV_1_, L, mean (SD)	1.35 (0.44)	1.34 (0.43)
Post-bronchodilator FEV_1_, % predicted, mean (SD)	48.2 (12.4)	48.1 (12.5)
Pre-bronchodilator FVC, L, mean (SD)	2.66 (0.83)	2.64 (0.80)
Pre-bronchodilator FVC, % predicted, mean (SD)	75.4 (18.0)	74.8 (17.9)
Post-bronchodilator FVC, L, mean (SD)	3.13 (0.90)	3.11 (0.86)
Post-bronchodilator FVC, % predicted, mean (SD)	88.7 (18.7)	88.2 (18.5)
Pre-bronchodilator FEV1/FVC ratio, %, mean (SD)	42.5 (10.3)	42.7 (10.4)
Post-bronchodilator FEV1/FVC ratio, %, mean (SD)	43.8 (10.5)	43.9 (10.7)
Smoking status		
Sustained ex-smoker, N (%)	1471 (59.7%)	1502 (58.2%)
Intermittent smoker, N (%)	679 (27.6%)	761 (29.5%)
Continuing smoker, N (%)	313 (12.7%)	316 (12.3%)
Pack-yrs smoking, mean (SD)	48.0 (27.9)	49.0 (28.0)
GOLD grade of airflow obstruction		
Grade I/II, N (%)	1179 (48.6%)	1226 (48.3%)
Grade III, N (%)	1059 (43.6%)	1118 (44.0%)
Grade IV, N (%)	189 (7.79%)	197 (7.75%)
Use of Inhaled Corticosteroids at baseline		
Yes	1506 (61.1%)	1581 (61.3%)
No	957 (38.9%)	998 (38.7%)
SGRQ total scores, mean (SD)	45.2 (17.2)	45.0 (17.0)

Table 2 shows the estimated average change per year over 4 years in the absolute bronchodilator response (Δ, in ml) (±SE) for FEV_1_ and FVC in the placebo arm both for all subjects and by GOLD grading for airflow obstruction, age, gender, smoking status and baseline ICS use. The estimated changes per year in FEV1 and FVC responses were significant for all subgroups, except for changes in FVC responses among the continuing smokers. The annual changes in the absolute FEV1 response were larger for sustained ex-smokers than continuing smokers (p = 0.0264) and for those receiving ICS at baseline (p = 0.0081) but did not differ significantly by GOLD grade of airflow obstruction, age or gender. The changes per year in FVC responses were significantly larger in GOLD III and IV compared to GOLD I/II (p = 0.0059 and 0.0006, respectively), in subjects >65 vs ≤ 65 yrs of age (0.0022), in sustained ex-smokers than continuing smokers (p = 0.0088), and in those receiving versus not receiving ICS at baseline (p < 0.0001). Similar data are shown in Additional file 2 for the tiotropium treatment group. For all subjects analyzed, results were comparable to those in the placebo group, although differences were noted in some of the subgroups.

**Table 2:** **Estimated average change per year over 4 years in absolute bronchodilator response (Δ, in ml) (±SE) for FEV**_**1**_ **and FVC in the placebo arm of the UPLIFT trial by GOLD grading for airflow obstruction (I&II, III, IV), age (≤50 yrs, >50 yrs), gender, and smoking status (sustained ex-smoker, intermittent smoker, continuing smoker)**

**Group**	**FEV**_**1**_		**FVC**	
	**Estimated change in Δ (SE) per yr**	**p value**	**Estimated change in Δ (SE) per yr**	**p value**
All	-10.9 (0.76)	<0.0001	-20.6 (1.87)	<0.0001
GOLD Stage				
I & II	-11.1 (1.10)	<0.0001	-14.4 (2.42)^1^	<0.0001
III	-11.2 (1.10)	<0.0001	-25.2 (3.08)	<0.0001
IV	-13.1 (2.35)	<0.0001	-40.6 (8.21)	<0.0001
Age, yrs				
≤65 yrs	-11.6 (1.05)	<0.0001	-15.5 (2.64)^2^	<0.001
>65 yrs	-10.1 (1.09)	<0.0001	-2.66 (2.60)	<0.0001
Gender				
Male	-10.7 (0.92)	<0.0001	-21.4 (2.27)	<0.0001
Female	-11.5 (1.21)	<0.0001	-18.4 (2.98)	<0.0001
Smoking status				
Sustained ex-smoker	-11.9 (0.93) ^3^	<0.0001	-24.6 (2.37)^3^	<0.0001
Intermittent smoker	-10.5 (1.48)	<0.0001	-17.3 (3.57)	<0.0001
Continuing smoker	-6.77 (2.42)	0.0053	-9.17 (5.70)	0.11
Inhaled steroids (baseline)				
No	-8.41 (1.24)^4^	<0.0001	-11.3 (3.01)^4^	0.0002
Yes	-12.5 (0.95)	<0.0001	-26.6 (2.38)	<0.0001

^1^Significantly different from GOLD III (p = 0.0059) and GOLD IV (p = 0.0006).

^2^Significantly different from age >65 yrs (pp = 0.0022).

^3^Signficiantly different from continuing smokers (p = 0.0264 for FEV1 and p = 0.0088 for FVC).

^4^Significantly different from those with baseline inhaled steroids (p = 0.0081 for FEV1 and p < 0.0001 for FVC)).

**Figure 2:**
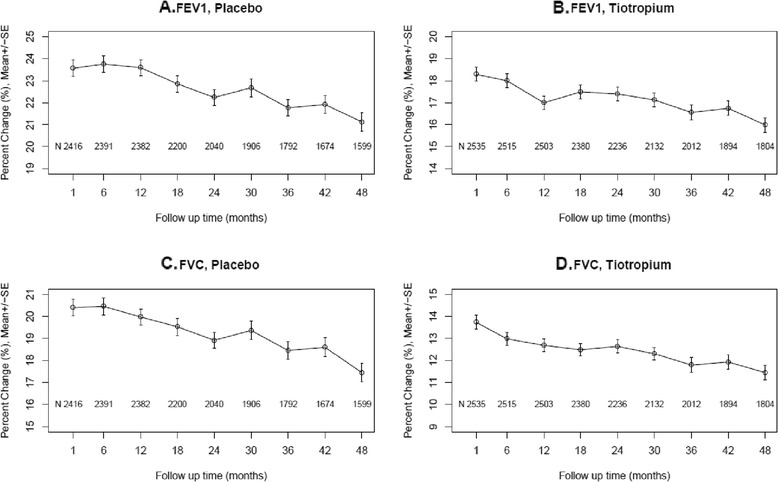
Mean percent changes (±SD) (Δ = post - minus pre-bronchodilator FEV1 and FVC, ml/pre-bronchodilator FEV1 and FVC, ml * 100, %) over 4 years by treatment group. Mean relative FEV1 responses (percent changes) are shown for the placebo group **(A)** and the tiotropium group **(B)** separately. Mean relative responses (percent changes) in FVC are shown for the placebo group **(C)** and the tiotropium group **(D)** separately.

The time trends of relative bronchodilator responses expressed as percent changes in FEV1 and FVC from the pre-bronchodilator values are shown for all subjects in each treatment group in Figure 2. Similar to the findings for absolute changes, the percent changes in both FEV1 and FVC declined progressively over 4 years, beginning at 1 to 1.5 years after trial initiation for all subjects in both treatment groups. Estimated average changes per year in relative bronchodilator responses for FEV1 and FVC (% ± SE) in all subjects and by GOLD stage, age, gender, smoking status and baseline ICS use are shown in Table 3 for the placebo group and Additional file 3 for tiotropium subjects. In placebo subjects, declines in both FEV1 and FVC responses were significantly greater in GOLD IV vs. both GOLD I/II and GOLD III, in sustained ex-smokers than continuing smokers and in those using ICS at baseline but did not differ by age or gender, except for significantly greater declines in FVC responses in older subjects. Similar findings were noted in tiotropium subjects.

**Table 3:** **Estimated average change per year over 4 years in relative bronchodilator response (percent change Δ = post- minus pre-bronchodilator FEV1 and FVC/pre-bronchodilator value X 100) (±SE) in the placebo arm by GOLD grading for airflow obstruction (I&II, III, IV), age (≤50 yrs, >50 yrs), gender, and smoking status (sustained ex-smoker, intermittent smoker, continuing smoker)**

**Group**	**FEV**_**1**_		**FVC**	
	**Estimated change in Δ (SE) per yr**	**p value**	**Estimated change in Δ (SE) per yr**	**p value**
All	-0.68 (0.08)	<0.0001	-0.66 (0.09)	<0.0001
GOLD Stage				
I & II	-0.40 (0.11)^1^	0.0001	-0.28 (0.10)^1^	0.0085
III	-0.86 (0.14)^2^	<0.0001	-0.89 (0.15)^2^	<0.0001
IV	-2.08 (0.38)	<0.0001	-2.11 (0.47)	<0.0001
Age, yrs				
≤65 yrs	-0.61 (0.11)	<0.0001	-0.34 (0.12)^3^	0.0058
>65 yrs	-0.77 (0.12)	<0.0001	-1.04 (0.13)	<0.0001
Gender				
Male	-0.61 (0.10)	<0.0001	-0.64 (0.10)	<0.0001
Female	-0.90 (0.17)	<0.0001	-0.74 (0.19)	<0.0001
Smoking status				
Sustained ex-smoker	-0.91 (0.11)^4^	<0.0001	-0.88 (0.11)^3^	<0.0001
Intermittent smoker	-0.54 (0.16)	0.0007	-0.55 (0.17)	0.0014
Continuing smoker	0.12 (0.25)	0.64	0.14 (0.26)	0.59
Inhaled steroids (baseline)				
No	-0.30 (0.13)^5^	0.0273	-0.13 (0.14)^5^	0.35
Yes	-0.93 (0.11)	<0.0001	1.01 (0.12)	<0.0001

^1^Significantly different from GOLD III (p = 0.0072 for FEV1 and p = 0.0008 for FVC) and GOLD IV (p < 0.0001 for FEV1 and p < 0.0001 for FVC).

^2^Significantly different from GOLD IV (p = 0.0018 for FEV1 and p = 0.0058 for FVC).

^3^Significantly different from age >65 (p < 0.0001).

^4^Signficiantly different from continuing smokers (p < 0.0001 for FEV1 and p = 0.0002 for FVC).

^5^Significantly different from those with baseline inhaled steroids (p = 0.0002 for FEV1 and p < 0.0001 for FVC).

**Figure 3:**
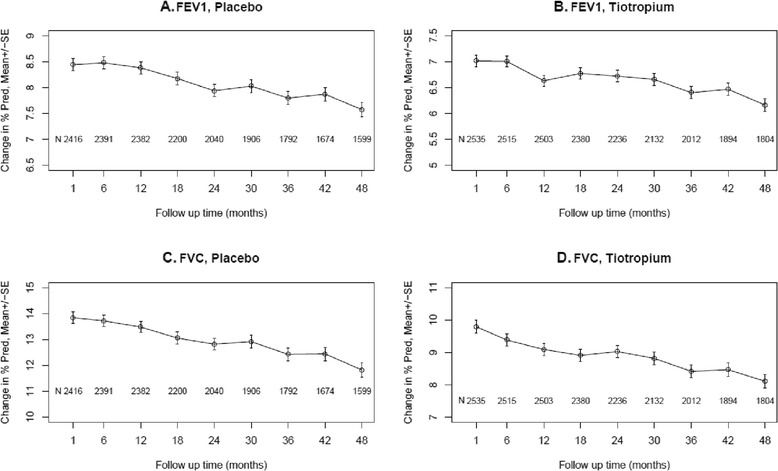
**Mean (±SD) % predicted (Δ = post-bronchodilator [% predicted] - minus pre-bronchodilator [% predicted] FEV1 and FVC) bronchodilator responses over 4 years by treatment group.** Mean % predicted FEV1 responses are shown for the placebo group (A) and the tiotropium group (B) separately. Mean percent predicted responses in FVC are shown for the placebo group (C) and the tiotropium group (D) separately.

FEV1 and FVC responses to the bronchodilators expressed as % predicted values over 4 years are shown for all patients in each treatment group at each time point in Figure 3. As with the other methods of expressing the bronchodilator response, a progressive decline in % predicted responses for both FEV1 and FVC over 4 years was observed in both treatment groups. Estimated average changes per year in % predicted responses for FEV1 and FVC for all subjects and by subgroups are shown in Table 4 for placebo subjects and Additional file 4 for tiotropium subjects. For all subjects in both treatment groups annual declines were modest but highly significant (p < 0.0001). In placebo subjects, declines in percent predicted FVC responses were significantly larger in GOLD IV and GOLD III than GOLD I/II subjects (p = 0.0013 and 0.0023, respectively) and in older than younger patients (p = 0.0040), declines in % predicted FEV1 responses were significantly larger in women than men (p < 0.05) and declines in both FEV1 and FVC % predicted responses were significantly greater in sustained ex-smokers than continuing smokers (p = 0.0267 and 0.0038, respectively), and in those on ICS at baseline (p = 0.0045 and p < 0.0001, respectively). Somewhat comparable findings were noted in the tiotropium group (Additional file 4).

**Table 4:** **Estimated average change per year over 4 years in % predicted bronchodilator response (Δ = % predicted post-bronchodilator minus % predicted pre-bronchodilator FEV1 or FVC) (±SE) in the placebo arm by GOLD grading for airflow obstruction (I&II, III, IV), age (≤50 yrs, >50 yrs), gender, and smoking status (sustained ex-smoker, intermittent smoker, continuing smoker)**

**Group**	**FEV**_**1**_		**FVC**	
	**Estimated change in Δ (SE) per yr**	**p value**	**Estimated change in Δ (SE) per yr**	**p value**
All	-0.33 (0.03)	<0.0001	-0.53 (0.05)	<0.0001
GOLD Stage				
I & II	-0.33 (0.04)	<0.0001	-0.35 (0.07)^1^	<0.0001
III	-0.35 (0.04)	<0.0001	-0.68 (0.09)	<0.0001
IV	-0.41 (0.08)	<0.0001	-1.05 (0.24)	<0.0001
Age, yrs				
≤65 yrs	-0.33 (0.04)	<0.0001	-0.39 (0.07)^2^	<0.0001
>65 yrs	-0.32 (0.04)	<0.0001	-0.69 (0.08)	<0.0001
Gender				
Male	-0.30 (0.03)^3^	<0.0001	-0.51 (0.06)	<0.0001
Female	-0.43 (0.06)	<0.0001	-0.61 (0.11)	<0.0001
Smoking status				
Sustained ex-smoker	-0.37 (0.03)^4^	<0.0001	-0.65 (0.07)^3^	<0.0001
Intermittent smoker	-0.31 (0.05)	<0.0001	-0.46 (0.10)	<0.0001
Continuing smoker	-0.18 (0.08)	0.0322	-0.16 (0.15)	0.29
Inhaled steroids (baseline)				
No	-0.23 (0.04)^5^	0.0273	-0.27 (0.09)^6^	0.0024
Yes	-0.39 (0.03)	<0.0001	-0.70 ((0.07)	<0.0001

^1^Significantly different from GOLD III (p = 0.0023) and GOLD IV (p = 0.0013).

^2^Significantly different from no baseline inhaled steroids (p = 0.0040).

^3^Significantly different from women (p = 0.0428).

^4^Signficiantly different from continuing smokers (FEV1 p = 0.0267; FVC p = 0.0038).

^5^Significantly different from no baseline inhaled steroids (p = 0.0045).

^6^Significantly different from no baseline inhaled steroids (p < 0.0001).

A progressive decline was also observed in the proportion of subjects in each treatment arm who fulfilled ATS/ERS criteria for a significant bronchodilator response, averaging 6-9% reduction in the proportion of subjects likely to exhibit a significant bronchodilator response per year (Table 5).

**Table 5:** **Between- and within-subjects variability in the annual change in bronchodilator response expressed as absolute, relative and percent predicted changes** ^**1**^

	**Response measured by absolute change in FEV (ml)**		**Response measured by absolute change in FVC (ml)**	
	**Tiotropium**	**Placebo**	**Tiotropium**	**Placebo**
Between-subject variability in annual change (square-root of variance)	15.2	15.3	30.2	44.5
Within-subject variability (square-root of variance)	109.2	107.9	244.7	252.0
	**Response measured by % change in FEV (ml)**		**Response measured by % change in FVC (ml)**	
	**Tiotropium**	**Placebo**	**Tiotropium**	**Placebo**
Between-subject variability in annual change (square-root of variance)	1.44	1.65	1.43	1.98
Within-subject variability (square-root of variance)	11.3	12.0	11.0	12.4
	**Response measured by absolute change in FEV (% predicted)**		**Response measured by absolute change in FVC (% predicted)**	
	**Tiotropium**	**Placebo**	**Tiotropium**	**Placebo**
Between-subject variability in annual change (square-root of variance)	0.55	0.54	0.89	1.26
Within-subject variability (square-root of variance)	3.93	3.88	7.04	7.23

^1^The variations were estimated from the linear mixed effects models.

Findings from the analysis of the relationship between individual declines in bronchodilator response and individual changes in health-related quality of life (assessed as total SGRQ score) over the course of the study in the placebo group indicated a modest but statistically significant inverse relationship between decline in FEV1 (but not FVC) response and change in SGRQ score, after adjustment for both baseline FEV1 % predicted and exacerbation frequency (≥2 versus <2) in the first year. For for each 1 ml decline in FEV1 response there was an estimated 0.12 unit increase in total SGRQ score (or for each 10 ml decline in FEV1 response there was an estimated 1.2 unit increase in SGRQ score) (data not shown).

Between- and within-subjects variability in the annual change in absolute, relative and % predicted responses over 4 years was quite large, as shown in Table 6 for FEV1 and FVC and each treatment arm separately. No significant differences were observed between treatment groups. Variability in annual changes in FVC response was approximately twice as large as that in FEV1 responses when expressed as absolute or % predicted changes, but was similar when calculated as relative changes.

**Table 6:** **Long-term trends in the proportion of subjects in each treatment arm who achieved a significant bronchodilator response for FEV**_**1**_ **, FVC and either FEV**_**1**_ **or FVC**

	**FEV**				**FVC**				**FEV or FVC**			
	**Tiotropium**		**Placebo**		**Tiotropium**		**Placebo**		**Tiotropium**		**Placebo**	
**Time**	**N**	**Proportion**	**N**	**Proportion**	**N**	**Proportion**	**N**	**Proportion**	**N**	**Proportion**	**N**	**Proportion**
1 m	2535	0.42	2416	0.55	2535	0.47	2416	0.62	2535	0.59	2416	0.73
6 m	2515	0.42	2391	0.53	2515	0.44	2391	0.63	2515	0.57	2391	0.72
1 yr	2503	0.39	2382	0.53	2503	0.43	2382	0.63	2503	0.55	2382	0.72
1.5 yr	2380	0.40	2200	0.51	2380	0.43	2200	0.62	2380	0.56	2200	0.71
2 yr	2236	0.39	2040	0.49	2236	0.43	2040	0.60	2236	0.56	2040	0.69
2.5 yr	2132	0.39	1906	0.50	2132	0.42	1906	0.61	2132	0.55	1906	0.70
3 yr	2012	0.36	1792	0.47	2012	0.39	1792	0.59	2012	0.52	1792	0.69
3.5 yr	1894	0.36	1674	0.47	1894	0.41	1674	0.59	1894	0.52	1674	0.68
4 yr	1804	0.35	1599	0.47	1804	0.39	1599	0.56	1804	0.51	1599	0.66
Estimated OR (95% CI) for change in proportion per year*	0.92 (0.90, 0.94)		0.91 (0.89, 0.94)		0.93 (0.91, 0.95)		0.94 (0.92, 0.96)		0.92 (0.90, 0.94)		0.92 (0.90, 0.95)	
p-value	<0.0001		<0.0001		<0.0001		<0.0001		<0.0001		<0.0001	

*The OR (odds ratio) and its 95% CI were estimated with GEE methods using data starting from 1 m.

FEV: Tiotropium vs. placebo p = 0.79.

FVC: Tiotropium vs. placebo p = 0.60.

FEV or FVC: Tiotropium vs. placebo p = 0.84.

## Discussion

We have shown that, in moderate to very severe COPD, mean responses to a near-maximal bronchodilator challenge decline progressively over time to a statistically significant, albeit modest and highly variable, extent, irrespective of the method of calculating the responses (absolute, relative or % predicted pre-post bronchodilator change). These downward trends in bronchodilator responses were observed in nearly all subgroups defined by the initial severity of airflow obstruction, age, gender, smoking status over the course of the study and use of ICS at baseline. However, the magnitude of the decline in responses for FEV1 or FVC differed significantly within some of these subgroups; for example, declines over time in absolute, relative and % predicted FEV1 and/or FVC responses were larger in patients with severe and very severe vs. mild/moderate airflow, > vs. ≤65 years of age, in sustained ex-smokers than continuing smokers and in patients receiving versus not receiving ICS at baseline (Tables 2, 3 and 4). The declines in responses for FVC tended to be larger than those for FEV1 when these were assessed as absolute changes, but not as relative or percent predicted changes. While some differences in the mean annual changes in FEV1 and FVC responses were noted between the placebo and tiotropium arms of the trial, these differences were not statistically significant irrespective of the method of expressing the bronchodilator response.

Responses to a bronchodilator in COPD patients are well known to vary over a relatively short time frame such that a large proportion of patients who respond significantly to a bronchodilator challenge on one day fail to do so on another day and vice versa over a relatively short time frame [4,5,15]. On the other hand, *long-term* trends in bronchodilator responses over more than one year have infrequently been measured [7,8]. In the IPPB trial, in which the average baseline pre-bronchodilator FEV1 (36.1% predicted) was comparable to that in UPLIFT (39.4% predicted), the mean change in the relative FEV1 response per year over 3 years (-0.58/yr) was similar to that which we observed in UPLIFT over 4 years (-0.68/yr) but, unlike the present findings, was not significantly different from zero [7]. On the other hand, the change in % predicted FEV1 response over 3 years (-0.36/yr) was both similar to that noted in the UPLIFT population over 4 years (-0.33/yr) and also significantly different from zero. The long-term change in *absolute* responses in the IPPB trial was not reported.

In contrast, in the 5-year LHS, in which the mean baseline pre-bronchodilator FEV1 (75.4% predicted) was much higher than that in either the IPPB trial or UPLIFT, a substantial increase in responsiveness (assessed as relative, absolute and % predicted responses) was noted over the first year, with either a slight further increase or no change over the ensuing 4 years and substantial *increases* from year 5 to year 11, except for the absence of any perceptible change only in absolute responses in sustained quitters over this time frame [8]. The reason for these disparate findings in the LHS compared to both the IPPP study and UPLIFT is unclear but might possibly be related to the much milder degree of airflow obstruction in LHS participants compared to those in the other two studies. The observation that the decline in relative responses in UPLIFT was significantly greater in patients with severe/very severe than mild/moderate airflow obstruction seems consistent with this possible explanation; however, even the subgroup of UPLIFT subjects with mild/moderate obstruction showed highly significant declines in both FEV1 and FVC responses, irrespective of the method of expressing these responses.

In this analysis, as in previous reports [5,7–9], the response to a bronchodilator has been shown to be highly variable both between and within individuals. Moreover, because of this variability and because responsiveness has not been shown to be predictive of exacerbations or mortality in ECLIPSE [9] or predict the long-term response to a bronchodilator over 1 year [16,17], bronchodilator responsiveness has been considered to be an unreliable phenotype [9]. However, the progressive decline in bronchodilator responses over time demonstrated in UPLIFT, as well as in the IPPB trial, mirrors to some extent the usually progressive, but admittedly variable, decline in lung function characteristic of COPD, suggesting that these two phenomena might be inter-related. One can only speculate as to the mechanism of the observed declines in bronchodilator responses over time. One possible mechanism is a progressive increase in the thickness of the walls of the small airways with progressive increases in the severity of airflow obstruction, as reported by Hogg et al. [18]; the resulting decreases in airway wall compliance could diminish the effect of bronchodilator-induced airway smooth muscle relaxation in increasing the patency of the lumen. It is also possible that the age-related loss of lung elastic recoil [19] that is most likely amplified in patients with progressive emphysema could counteract drug-induced bronchodilation and reduction in air-trapping by increasing dynamic airway compression.

One clinical implication of the progressive decline in bronchodilator responses over multiple years is that this might result in a reduced effectiveness of bronchodilator therapy on clinical outcomes in COPD as the disease progresses over time, at least in some patients in view of the large inter-individual variability observed in the decline in responsiveness. The finding of a modest but statistically significant within-individual relationship between declines in acute bronchodilator responses on the one hand and worsening SGRQ scores on the other suggests that decrements in the response to a bronchodilator over time might be associated with poorer clinical outcomes, although this association does not necessarily imply causality. Moreover, the only modest changes in SGRQ score in association with declines in bronchodilator response (1.2 unit increase in SGRQ for each 10 ml decline in acute FEV1 response) argue against a clinically meaningful relationship. Another implication of our findings is that the decline in the acute bronchodilator response over time would lead to a partial convergence of the slopes of decline in lung function calculated from the pre- and post-bronchodilator FEV1 (and FVC), resulting in a steeper post- than pre-bronchodilator slope [10]. Moreover, these potential consequences are likely to be relatively independent of the severity of airflow obstruction, age, gender and smoking status since significantly progressive declines in bronchodilator responses were seen in most of these subgroups. The possible impact on the slope of change in post- vs. pre-bronchodilator lung function over time needs to be taken into account in the design of long-term trials in which the annual decline in post-bronchodilator lung function is measured as a means of assessing the rate of progression of COPD.

The strengths of the present analysis include the large number of patients with varying degrees of severity of airflow obstruction who were followed over an extended period of time, the high quality and reproducibility of spirometry [14] and the relatively large doses of the two different classes of short-acting bronchodilators that were administered along with the timing of post-bronchodilator spirometry to coincide with the time of expected peak action of each class of bronchodilator.

A major limitation is the large drop-out rate with only 27%, 35% and 40% of subjects completing visits at 2, 3 and 3¾ yrs, respectively. The current analysis uses all available data and the statistical inference from linear mixed effects models is valid under the missing at random assumption. Moreover, those who discontinued the trial prematurely were more likely to have fared poorly during the trial, suggesting that their bronchodilator responses, had they been measured subsequent to their withdrawal, might tend to be even less robust than the responses at later time points in those subjects who completed the trial.

In conclusion, acute responses of both FEV1 and FVC to near maximal doses of two different bronchodilators, while considerably variable both between and within individuals, on average diminish progressively and significantly over time, consistent with the usually progressive decline in lung function with age in patients with COPD. These declines were independent of the method of expressing the bronchodilator response and tended to be larger in patients with severe/very severe compared to those with mild/moderate airflow obstruction, in patients >65 years of age and in former than continuing smokers and in those not on ICS at baseline. These declines in the response to a bronchodilator imply a possible diminution in the clinical efficacy of bronchodilator therapy over time and may account for differences in the slopes of lung function decline with age when calculated using the post- compared to the pre-bronchodilator value.

## Additional files

Additional file 1:
**Duration of time medication to be withheld prior to each clinic visit.**
Additional file 2:
**Estimated average change per year over 4 years in absolute bronchodilator response (**Δ**, in ml) (±SE) for FEV**_**1**_ **and FVC in the tiotropium arm of the UPLIFT trial by GOLD grading for airflow obstruction (I&II, III, IV), age (≤50 yrs, >50 yrs), gender, and smoking status (sustained ex-smoker, intermittent smoker, continuing smoker).**
Additional file 3:
**Estimated average change per year over 4 years in relative bronchodilator response (percent change [** **Δ] = post- minus pre-bronchodilator FEV1 and FVC/pre-bronchodilator value X 100) (±SE) in the tiotropium arm by GOLD grading for airflow obstruction (I&II, III, IV), age (≤50 yrs, >50 yrs), gender, and smoking status (sustained ex-smoker, intermittent smoker, continuing smoker).**
Additional file 4:
**Estimated average change per year over 4 years in % predicted bronchodilator response (Δ= % predicted post-bronchodilator minus % predicted pre-bronchodilator FEV1 or FVC) l) (±SE) in the tiotropium arm of the trial by GOLD grading for airflow obstruction (I&II, III, IV), age (≤50 yrs, >50 yrs), gender, and smoking status (sustained ex-smoker, intermittent smoker, continuing smoker).**

## Abbreviations

COPD: Chronic obstructive pulmonary disease
FEV1: Forced expired volume in 1 second
FVC: Forced vital capacity
UPLIFT: Understanding Potential Long-term Impacts on Function with Tiotropium
IPPB: Intermittent positive pressure breathing
LHS: Lung Health Study
ECLIPSE: Evaluation of COPD Longitudinally to Identify Predictive Surrograte Endpoints
