# Supplementary material for: Acute bronchodilator responses decline progressively over 4 years in patients with moderate to very severe COPD
Source: Respir Res. 2014 Aug 31;15(1):102. doi: 10.1186/s12931-014-0102-5 (PMC4244051; doi:10.1186/s12931-014-0102-5)
Supplement: Additional file 1: — Duration of time medication to be withheld prior to each clinic visit. [file 12931_2014_102_MOESM1_ESM.doc]

**Additional file 1**: Duration of time medication to be withheld prior to each clinic visit

| **Medication** | **Duration medication to be withheld** |
| --- | --- |
| Study drug | 24 hrs |
| Short-acting beta-agonist | 8 hrs |
| Long-acting beta-agonist (either alone or in a fixed-dose with an inhaled corticosteroid) | 12 hrs |
| Inhaled corticosteroid (either as a mono-product or in a fixed combination with a long-acting beta-agonist | 12 hrs |
| Twice-daily theophylline | 24 hrs |
| Once-daily theophylline | 48 hrs |
