# Supplementary material for: Acute bronchodilator responses decline progressively over 4 years in patients with moderate to very severe COPD
Source: Respir Res. 2014 Aug 31;15(1):102. doi: 10.1186/s12931-014-0102-5 (PMC4244051; doi:10.1186/s12931-014-0102-5)
Supplement: Additional file 2: — Estimated average change per year over 4 years in absolute bronchodilator response (Δ, in ml) (±SE) for FEV 1 and FVC in the tiotropium arm of the UPLIFT trial by GOLD grading for airflow obstruction (I&II, III, IV), age (≤50 yrs, >50 yrs), gender, and smoking status (sustained ex-smoker, intermittent smoker, continuing smoker). [file 12931_2014_102_MOESM2_ESM.doc]

**Additional file 2.** Estimated average change per year over 4 years in absolute bronchodilator response (Δ, in ml) (±SE) for FEV_1_ and FVC in the tiotropium arm of the UPLIFT trial by GOLD grading for airflow obstruction (I&II, III, IV), age (≤50 yrs, >50 yrs), gender, and smoking status (sustained ex-smoker, intermittent smoker, continuing smoker)

| **Group** | **FEV_1_** | | **FVC** | |
| --- | --- | --- | --- | --- |
|  | **Estimated change in Δ (SE) per yr** | **p value** | **Estimated change in Δ (SE) per yr** | **p value** |
| All | -8.90 (0.72) | <0.0001 | -17.0 (1.58) | <0.0001 |
| GOLD Stage |  |  |  |  |
| I & II | -7.33 (1.12)^1^ | <0.0001 | -8.18 (2.18)^1^ | 0.0002 |
| III | -10.8 (0.97) | <0.0001 | -24.8 (2.45) | <0.0001 |
| IV | -10.1 (2.34) | <0.0001 | -30.4 (6.55) | <0.0001 |
| Age, yrs |  |  |  |  |
| ≤65 yrs | -9.40 (1.05) | <0.0001 | -13.8 (2.20)^2^ | <0.0001 |
| >65 yrs | -8.34 (0.97) | <0.0001 | -21.0 (2.25) | <0.0001 |
| Gender |  |  |  |  |
| Male | -9.48 (0.88) | <0.0001 | -19.1 (1.92)^3^ | <0.0001 |
| Female | -7.08 (1.16) | <0.0001 | -10.5 (2.51) | <0.0001 |
| Smoking status |  |  |  |  |
| Sustained ex-smoker | -8.96 (0.90) | <0.0001 | -16.7 (1.96)^4^ | <0.0001 |
| Intermittent smoker | -9.84 (1.35) | <0.0001 | -21.7 (3.01) | <0.0001 |
| Continuing smoker | -6.07 (2.41) | 0.0125 | -6.14 (5.21) | 0.24 |
| Inhaled steroids (baseline) |  |  |  |  |
| No | -6.68 (1.22)^5^ | <0.0001 | -9.50 (2.69)^5^ | 0.0004 |
| Yes | -10.3 (0.89) | <0.0001 | -21.7 (1.94) | <0.0001 |

^1^Significantly different from GOLD III (p=0.0192 for FEV1 and p<0.0001 for FVC) and, for FVC only, from GOLD IV (p=0.0005)

^2^Signficiantly different from older subjects (p=0.0231)

^3^Significantly different from female subjects (p=0.02)

^4^Significantly different from continuing smokers (p=0.0289)

Significantly different from those with baseline inhaled steroids (p=0.0138 for FEV1 and p=0.0001 for FVC)
