# Supplementary material for: Acute bronchodilator responses decline progressively over 4 years in patients with moderate to very severe COPD
Source: Respir Res. 2014 Aug 31;15(1):102. doi: 10.1186/s12931-014-0102-5 (PMC4244051; doi:10.1186/s12931-014-0102-5)
Supplement: Additional file 3: — Estimated average change per year over 4 years in relative bronchodilator response (percent change [ Δ] = post- minus pre-bronchodilator FEV1 and FVC/pre-bronchodilator value X 100) (±SE) in the tiotropium arm by GOLD grading for airflow obstruction (I&II, III, IV), age (≤50 yrs, >50 yrs), gender, and smoking status (sustained ex-smoker, intermittent smoker, continuing smoker). [file 12931_2014_102_MOESM3_ESM.doc]

**Additional file 3.** Estimated average change per year over 4 years in relative bronchodilator response (percent change [Δ] = post- minus pre-bronchodilator FEV1 and FVC/pre-bronchodilator value X 100) (±SE) in the tiotropium arm by GOLD grading for airflow obstruction (I&II, III, IV), age (≤50 yrs, >50 yrs), gender, and smoking status (sustained ex-smoker, intermittent smoker, continuing smoker)

| **Group** | **FEV_1_** | | **FVC** | |
| --- | --- | --- | --- | --- |
|  | **Estimated change in Δ (SE) per yr** | **p value** | **Estimated change in Δ (SE) per yr** | **p value** |
| All | -0.56 (0.07) | <0.0001 | -0.52 (0.07) | <0.0001 |
| GOLD Stage |  |  |  |  |
| I & II | -0.17 (0.10)^1^ | 0.092 | -0.08 (0.09)^1^ | 0.35 |
| III | -0.88 (0.11) | <0.0001 | -0.83 (0.12) | <0.0001 |
| IV | -1.40 (0.34) | <0.0001 | -1.28 (0.38) | 0.0008 |
| Age, yrs |  |  |  |  |
| ≤65 yrs | -0.53 (0.10) | <0.0001 | -0.34 (0.12)^2^ | 0.0058 |
| >65 yrs | -0.60 (0.11) | <0.0001 | -1.04 (0.13) | <0.0001 |
| Gender |  |  |  |  |
| Male | -0.60 (0.08) | <0.0001 | -0.37 (0.10) | <0.0001 |
| Female | -0.42 (0.16) | 0.0078 | -0.69 (0.10) | 0.0177 |
| Smoking status |  |  |  |  |
| Sustained ex-smoker | -0.67 (0.09)^3^ | <0.0001 | -0.55 (0.09)^3^ | 0.0002 |
| Intermittent smoker | -0.55 (0.14) | 0.0001 | -0.62 (0.14) | <0.0001 |
| Continuing smoker | -0.02 (0.22) | 0.64 | -0.06 (0.23) | 0.78 |
| Inhaled steroids (baseline) |  |  |  |  |
| No | -0.31 (0.12)^4^ | 0.0142 | -0.17 (0.13)^4^ | 0.18 |
| Yes | -0.72 (0.09) | <0.0001 | -0. 73 (0.09) | <0.0001 |

^1^Significantly different from GOLD III (p<0.0001 for both FEV1 and FVC) and from GOLD IV (p<0.0001 for both FEV1 and FVC)

^2^Signficiantly different from older subjects (<0.0001)

^3^Significantly different from continuing smokers (p=0.0038 for FEV1 and p=0.0269 for FVC)

^4^Significantly different from those with baseline inhaled steroids (p=0064 for FEV1 and p=0.0001 for FVC)
