# Supplementary material for: Acute bronchodilator responses decline progressively over 4 years in patients with moderate to very severe COPD
Source: Respir Res. 2014 Aug 31;15(1):102. doi: 10.1186/s12931-014-0102-5 (PMC4244051; doi:10.1186/s12931-014-0102-5)
Supplement: Additional file 4: — Estimated average change per year over 4 years in % predicted bronchodilator response (Δ= % predicted post-bronchodilator minus % predicted pre-bronchodilator FEV1 or FVC) l) (±SE) in the tiotropium arm of the trial by GOLD grading for airflow obstruction (I&II, III, IV), age (≤50 yrs, >50 yrs), gender, and smoking status (sustained ex-smoker, intermittent smoker, continuing smoker). [file 12931_2014_102_MOESM4_ESM.doc]

**Additional file 4.** Estimated average change per year over 4 years in % predicted bronchodilator response (Δ= % predicted post-bronchodilator minus % predicted pre-bronchodilator FEV1 or FVC) l) (±SE) in the tiotropium arm of the trial by GOLD grading for airflow obstruction (I&II, III, IV), age (≤50 yrs, >50 yrs), gender, and smoking status (sustained ex-smoker, intermittent smoker, continuing smoker)

| **Group** | **FEV_1_** | | **FVC** | |
| --- | --- | --- | --- | --- |
|  | **Estimated change in Δ (SE) per yr** | **p value** | **Estimated change in Δ (SE) per yr** | **p value** |
| All | -0.26 (0.03) | <0.0001 | -0.42 (0.05) | <0.0001 |
| GOLD Stage |  |  |  |  |
| I & II | -0.19 (0.04)^1^ | <0.0001 | -0.16 (0.06)^1^ | 0.0128 |
| III | -0.34 (0.03) | <0.0001 | -0.65 (0.07) | <0.0001 |
| IV | -0.35 (0.07) | <0.0001 | -0.83 (0.18) | <0.0001 |
| Age, yrs |  |  |  |  |
| ≤65yrs | -0.26 (0.04) | <0.0001 | -0.30 (0.06)^2^ | <0.0001 |
| >65 yrs | -0.26(0.04)^2^ | <0.0001 | -0.56 (0.07) | <0.0001 |
| Gender |  |  |  |  |
| Male | -0.27 (0.03) | <0.0001 | -0.46 (0.05) | <0.0001 |
| Female | -0.24 (0.05) | <0.0001 | -0.30 (0.10) | 0.0029 |
| Smoking status |  |  |  |  |
| Sustained ex-smoker | -0.27 (0.03) | <0.0001 | -0.42 (0.06)^3^ | <0.0001 |
| Intermittent smoker | -0.28 (0.05) | <0.0001 | -0.56 (0.09) | <0.0001 |
| Continuing smoker | -0.16 (0.08) | 0.0501 | -0.06 (0.14) | 0.68 |
| Inhaled steroids (baseline) |  |  |  |  |
| No | -0.18 (0.04)^4^ | <0.0001 | -0.20 (08)^4^ | 0.0099 |
| Yes | -0.31 (0.03) | <0.0001 | -0.55 (0.06) | <0.0001 |

^1^Significantly different from GOLD III (p=0.0036 for FEV1 and p<0.0001 for FVC) and, for FVC only, from GOLD IV (p=0.0004)

^2^Signficiantly different from older subjects (p=0.0049)

^3^Significantly different from continuing smokers (p=0.0091)

Significantly different from those with baseline inhaled steroids (p=0.0123 for FEV1 and p=0.0001 for FVC)
